# Proteomics-Based Mechanistic Investigation of *Escherichia coli* Inactivation by Pulsed Electric Field

**DOI:** 10.3389/fmicb.2019.02644

**Published:** 2019-11-08

**Authors:** Zhenyu Liu, Lingying Zhao, Qin Zhang, Nan Huo, Xiaojing Shi, Linwei Li, Liyan Jia, Yuanyuan Lu, Yong Peng, Yanbo Song

**Affiliations:** ^1^Information Science and Engineering College, Shanxi Agricultural University, Jinzhong, China; ^2^Department of Food, Agricultural and Biological Engineering, The Ohio State University, Columbus, OH, United States; ^3^Life Science College, Shanxi Agricultural University, Jinzhong, China; ^4^College of Food Science and Engineering, Shanxi Agricultural University, Jinzhong, China; ^5^Shanghai Applied Protein Technology Co., Ltd., Shanghai, China

**Keywords:** pulsed electric field, *E. coli*, proteomics, cell inactivation, molecular mechanisms

## Abstract

The pulsed electric field (PEF) technology has been widely applied to inactivate pathogenic bacteria in food products. Though irreversible pore formation and membrane disruption is considered to be the main contributing factor to PEF’s sterilizing effects, the exact molecular mechanisms remain poorly understood. In this study, by using mass spectrometry (MS)-based label-free quantitative proteomic analysis, we compared the protein profiles of PEF-treated and untreated *Escherichia coli*. We identified a total of 175 differentially expressed proteins, including 52 candidates that were only detected in at least two of the three samples in one experiment group but not in the other group. Functional analysis revealed that the differential proteins were primarily involved in the regulation of cell membrane composition and integrity, stress response, as well as various metabolic processes. Quantitative reverse-transcription polymerase chain reaction (qRT-PCR) analysis was conducted on the genes of selected differential proteins at varying PEF intensities, which were known to result in different cell killing levels. The qRT-PCR data confirmed that the proteomic results could be reliably used for further data interpretation, and that the changes in the expression levels of the differential candidates were, to a large extent, caused directly by the PEF treatment. The findings of the current study offered valuable insight into PEF-induced cell inactivation.

## Introduction

The pulsed electric field (PEF) technology is a mild, non-thermal method that processes various biological materials with intermittent high-intensity electric energy (Clark, 2006). Compared to other thermal processing techniques, PEF generates substantially less heat, which ensures better preservation of flavor, color and nutrition in food products. PEF also offers obvious advantages over both enzyme- and chemical-based cold processing methods in that it does not introduce any potentially quality-degrading additives. One of the main applications of PEF in food and beverage industry is the inactivation of microbial pathogens. For example, liquid whole egg processed by combination of PEF and mild thermal treatment at 55°C exhibited significantly longer shelf-life at 4°C than those treated with heat alone (Hermawan et al., 2004). In another study, *Listeria* monocytogenes populations in different liquid milk products were reduced by a factor of 10^4^ with a 0.6-ms PEF treatment at 50°C (Reina et al., 1998). Recently, PEF has also been evaluated for the preservation of solid food products, such as fruits (Sotelo et al., 2015; Yu et al., 2017), and for facilitating the extraction of valuable chemicals from plants and fungi (Goettel et al., 2013; Xue and Farid, 2015).

Currently, studies that aim to investigate the mechanism of PEF-induced cell inactivation have focused predominantly on its deleterious effects on membrane integrity (Unal et al., 2002; Wu, 2008). It is generally accepted that cell membranes exposed to PEF are locally distorted by electromechanical compression, culminating in pore formation and even membrane rupture (Weaver and Chizmadzhev, 1996). In comparison, there have been very few studies on whether PEF also affects other cellular components or processes, particularly proteins (Li et al., 2011; Rivas et al., 2013). In this regard, it has been demonstrated that PEF could induce significant structural changes in solid-state egg white proteins (Qian et al., 2016). Meanwhile, PEF-processed malting barley seeds showed reduced α-amylase expression compared to the untreated controls (Dymek et al., 2012). In recent years, the rapid development of proteomic technologies has allowed researchers to systematically analyze proteome-wide changes in response to external stimuli. For example, Rivas et al. (2013) examined the proteomic changes in PEF-damaged *Escherichia coli* DH5α cells with or without a 1-h recovery in Luria-Bertani (LB) medium after the treatment. The recovery and non-recovery groups showed different sets of differentially expressed proteins, which were mainly associated with cell metabolism, membrane structure, aerobic respiration, and protein folding (Rivas et al., 2013). There have also been a number of other studies regarding the effects of PEF treatment or electroporation on the mRNA and/or protein expression in various prokaryotic and eukaryotic cells (Mlakar et al., 2009; Heller et al., 2010; Zhao et al., 2018). However, further research is needed in order to provide a more comprehensive and detailed elucidation of how PEF disrupts various aspects of cellular structures and functions to achieve its sterilizing effects.

## Materials and Methods

### Cell Culture and PEF Treatment

**FIGURE 1:**
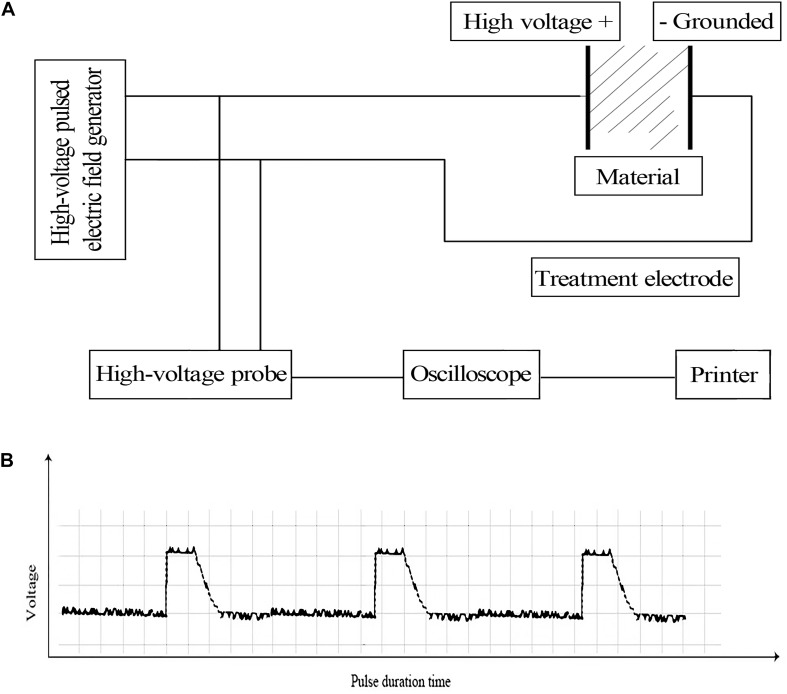
**(A)** Schematic diagram of the PEF device setup (Liu Z.Y. et al., 2016) and **(B)** wave shape of the pulse.

The *E. coli* strain CGMCC44102 was obtained from the China General Microbiological Culture Collection Center (CGMCCC), and cultured in LB medium at 150 rpm and 37°C to a final optical density (OD) of 1.0 at 600 nm. Subsequently, 1 mL of the culture broth was collected and centrifuged at 8000 rpm, 4°C for 2 min. The cell pellet was re-suspended in 2 mL of ice-cold 0.1% (w/v) bacteriological peptone solution (Solarbio Life Science, China) and divided into 70 μL aliquots. For PEF treatment, each aliquot was pipetted into a new, sterile 1.0-mm electroporation cuvette and pulsed using a BTX ECM830 Square Wave Electroporation System (BTX, United States) based on a protocol described previously (Liu Z.Y. et al., 2016). The cell density in the aliquot was approximately 10^8^ CFU/mL and electric conductivity was 0.19 mS/cm. The general setup of the device and the wave shape of the pulse were illustrated in Figure 1. After treatment, the cells were serially diluted and counted based on a previously described protocol (Aronsson et al., 2005) to calculate the inhibition rate. To achieve different cell killing levels, we employed different sets of pulse conditions as previously described. For a cell killing extent of 95 ± 2.0%, pulse intensity, number and duration were set to 14.5 kV⋅cm^–1^, 26 and 67 μs, respectively. The killing extent was 51 ± 1.3% with the treatment parameters of 6.10 kV⋅cm^–1^, pulse number of 54, pulse duration of 77 μs. And when the pulse intensity was 2.88 kV⋅cm^–1^, pulse number was 62, pulse duration was 82 μs, the killing extent was 29 ± 0.7%. Based on measurement on an RC05 Infrared Thermal Imaging Camera (Rinch Industrial, China), the culture temperature showed an average increase of less than 3°C following the PEF treatment, which would not significantly reduce the viability of the cells.

The electroporated aliquots were pooled together to a final volume of 3 mL (∼43 aliquots) and divided in a clean 1.5 mL microcentrifuge tube placed on ice. This was used as an experimental replicate. The pooled *E. coli* suspension was then centrifuged at 4°C, 5,000 × *g* for 5 min, washed twice with and then re-suspended in ice-cold phosphate-buffered saline (PBS), and then quickly flash-frozen in liquid nitrogen for storage at −80°C. For controls, a mock treatment without the application of PEF was performed, with identical downstream procedures. The whole experiment described above was conducted in triplicate.

### Protein Extraction

The cell pellet of strain was suspended in 200 μL SDT lysis buffer consisting of 4% (w/v) sodium dodecyl sulfate (SDS), 0.15 M Tris–HCl at pH 7.6 and 100 mM dithiothreitol (DTT), and transferred to a 2-mL tube pre-filled with 1/3 vol of quartz sand and ten steel grinding beads. The resultant mixture was subsequently homogenized twice on a Fastprep-24 homogenizer (MP Biochemicals, United States) at 6.0 m/s, followed by ten cycles of 10-s sonication at 80 W with 15-s intervals. The homogenate was boiled for 15 min and centrifuged at 14,000 × *g* for 40 min, after which the supernatant was sterilized with a 0.22-μm filter. Total protein in the filtrate was quantified with a BCA Protein Assay Kit (Bio-Rad, United States). All filtered protein extracts were stored at −80°C until use.

### Trypsin Digestion

Based on the BCA assay results, 200 μg of the extracted protein were denatured with 30 μL of SDT buffer and incubated at 56°C for 30 min. Low-molecular-weight components, including SDS and DTT, were removed by repeated ultrafiltration through a Microcon 10-kDa Centrifugal Filter Unit (Millipore, United States) with UA buffer, which consisted of 8 M urea in 150 mM Tris–HCl at pH 8.0, as the wash buffer. The sample was mixed with 100 μL of 100 mM iodoacetamide in UA, followed by a 30-min incubation in darkness. The filter unit was subsequently washed with 100 μL of UA twice and 100 μL of 25 mM NH_4_HCO_3_ twice. The resultant protein sample was digested by adding 4 μg of trypsin (Promega, United States) in 40 μL of 25 mM NH_4_HCO_3_ to the filter unit and then incubating at 37°C for 18 h. The filter unit was centrifuged at 13,400 rpm for 30 min and the peptide products were collected as a flow-through. The filter unit was next washed with 40 μL of 25 mM NH_4_HCO_3_ and centrifuged as above. The two flow-through were combined, desalted on an Empore C-18 Standard Density Solid-Phase Extraction Cartridge (bed I.D. 7 mm, volume 3 mL; Sigma-Aldrich, United States) and concentrated by vacuum centrifugation, followed by reconstituting the peptides in 40 μL of 0.1% (v/v) formic acid. Peptide quantification was performed by measuring the absorbance at 280 nm on an ultraviolet-visible spectrophotometer.

### Liquid Chromatography (LC)-MS/MS Analysis

Proteomic analysis was performed on an Easy-nLC 1,000 System (Thermo Fisher Scientific, United States) coupled to a Q Exactive HF Quadrupole-Orbitrap Mass Spectrometer (Thermo Fisher Scientific, United States). Each peptide sample was loaded onto an Acclaim PepMap100 C18 reverse-phase trap column (100 μm × 2 cm, nanoViper fitting; Thermo Fisher Scientific, United States) connected to an Easy C18 reverse-phase analytical column (75 μm × 10 cm, 3 μm resin; Thermo Fisher Scientific, United States) pre-equilibrated in buffer A [0.1% (v/v) formic acid]. The peptides were separated with a linear gradient of buffer B [84% (v/v) acetonitrile and 0.1% (v/v) formic acid] at a constant flow rate of 300 nL/min. The gradient of B was first increased from 0 to 55% over 110 min, then to 100% over 5 min, before being maintained at 100% for another 5 min.

Following the separation, the eluted peptides were immediately analyzed under positive-ion mode with peptide recognition enabled. The ions were first subjected to one survey scan in the m/z range of 300–1,800 at a mass resolution of 70,000 at m/z 200. Automatic gain control target, maximum inject time and dynamic exclusion were set to 3 × 10^6^, 10 ms and 40.0 s, respectively. MS2 spectra were obtained by using a data-dependent top 10 method to dynamically select the most abundant precursor ions from the survey scans for high-energy collisional dissociation (HCD) fragmentation. The resolution of the MS2 scans was set to 17,500 at m/z 200. Isolation width, normalized collision energy and underfill ratio were set to 2 m/z, 30 eV and 0.1%, respectively.

### Protein Identification and Quantification

The raw mass spectrometry (MS) data for each sample were combined and imported into MaxQuant (version 1.5.3.17, Max Planck Institute of Biochemistry, Germany) (Cox and Mann, 2008). For protein identification, the MS data were searched against the Uniprot *E. coli* protein database represented by the file uniprot_*Escherichia_coli*_1124415_20180910.fasta. Software settings were adjusted as follows: enzyme-trypsin; max missed cleavage-2; fixed modification-carbamidomethyl (C); variable modification-oxidation (M), acetyl (Protein N-term); main search ppm-6; MS/MS tolerance ppm-20; database pattern-reverse; include contaminants-true; false discovery rate (FDR) threshold-0.01; peptides for quantification-unique + razor; match between runs-2 min; protein quantification-label-free quantification (LFQ) (Cox et al., 2014); minimum ratio count-1.

Protein quantification was performed based on the LFQ algorithm as previously described (Cox et al., 2014; Liu et al., 2018). Intensity-based absolute quantification (iBAQ) in MaxQuant was performed on the identified peptides to quantify protein abundance. The significance of the differentially expressed proteins between the samples was examined with the cutoff values fold change >1.5 and *p* ≤ 0.05 by *t*-test.

### Bioinformatics Analysis

For gene ontology (GO^1^) analysis, the sequences of differentially expressed proteins were batch-searched against a local SwissProt *E. coli* protein database using the NCBI BLAST + client software (version 2.2.28) to identify any functionally annotated homologs. These annotations were then transferred and combined with those of the corresponding query *E. coli* proteins. To reduce the workload, only the top 10 blast hits of each query sequence with an E-value below 1 × 10^–3^ were selected. These candidates were annotated by Blast2GO (version 3.3.5; Stefan et al., 2008) with an E-value filter of 1 × 10^–6^, GO weight of 5, annotation cutoff of 75, and default gradual EC weights. Sequences that could not be annotated under these settings were then subjected a new round of annotation using more permissive parameters. Subsequently, the sequences that could not be annotated and those without BLAST hits were then searched against database by using InterProScan (Sun et al., 2016) to retrieve functional annotations of their conserved motifs. The combined GO annotation results were plotted by R scripts. For Kyoto Encyclopedia of Genes and Genomes (KEGG) pathway annotation, the sequences of differentially expressed proteins were searched via BLAST against the KEGG Database at https://www.genome.jp/kegg/kaas (Moriya et al., 2007).

Next, enrichment analyses were conducted using Fisher’s exact test in order to unearth the cellular and physiological roles of the differentially expressed proteins. The GO terms were categorized into three subcategories, including biological process, molecular function and cellular component. The Benjamini–Hochberg method was used to calculate the adjusted *p*-values. *p* < 0.05 was considered statistically significant.

### Hierarchical Clustering

Hierarchical clustering was performed to reveal global differences in protein expression profiles between the PEF treatment group and the control group. To this end, the data from protein relative expression were analyzed by Cluster 3.0 (Lu et al., 2012) using the average-linkage method for clustering, with similarity between genes expressed in Euclidean distance. The obtained dendrogram was visualized in Java Treeview (version 3.0) (Zhu et al., 2014). In addition, a heat map was also generated as a visual aid.

### Protein-Protein Interaction (PPI) Network

The differentially expressed proteins were imported into STRING to analyze their potential interactions (version 10.5) (Zhou et al., 2012). The results were downloaded in XGMML format and visualized in the form of an interaction network by importing into Cytoscape (version 3.2.1) (Zhu et al., 2014). The degree value of each protein node was calculated to evaluate its importance in the PPI network.

### Parallel Reaction Monitoring (PRM) Analysis

Parallel reaction monitoring analysis of selected proteins were conducted as previously described (Qu et al., 2018). Briefly, digested peptides were prepared as elucidated above. Then, 2 μg of the peptides were spiked with 20 fmol standard (PRTC: GISNEGQNASIK) and separated on an Easy nLC 1,200 System (Thermo Fisher Scientific, United States) with the same buffer A, B and flow rate as above. The gradient of B was set as follows: 5–10% over 2 min, 10–30% over 43 min, 30–100% over 10 min and then 100% for another 5 min. The eluted peptides were immediately analyzed under positive-ion mode on a Q-Exactive HF mass spectrometer (Thermo Fisher Scientific, United States). The ions were first subjected to one survey scan in the m/z range of 300 to 1,800 at a mass resolution of 60,000 at m/z 200. Automatic gain control target and maximum inject time were set to 3 × 10^6^ and 200 ms, respectively. The full MS1 scan were followed by 20 MS2 scans at a resolution of 17,500 at m/z 200 (Liu et al., 2019). Precursor ions were fragmented via the HCD method at normalized collision energy of 27 eV. Isolation window and maximum injection time were set to 1.6 Th and 120 ms, respectively. The obtained raw PRM data were imported into Skyline (version 3.5.0) (MacLean et al., 2010) for analysis.

### Quantitative Reverse-Transcription Polymerase Chain Reaction (qRT-PCR) Analysis

Following the PEF treatment, total RNA was immediately extracted using anRNAiso Plus Kit (TaKaRa, Japan), followed by reverse transcription with a PrimeScript Reverse Transcriptase Kit (TaKaRa, Japan). All gene-specific primers were designed using Primer Premier 5.0 software (Premier Biosoft International, United States) and synthesized by Sangon Biotech, China. Gene expression levels were analyzed by qRT-PCR using SYBR Green qPCR Mix (TaKaRa, United States) on a CFX96 Real-Time PCR System (Bio-Rad, United States) according to the manufacturers’ instructions. Glucan biosynthesis protein G (mdoG) was used as a control (Heng et al., 2011). All reactions were performed with at least three replicates. Fold changes were calculated based on the 2^–ΔΔ^*^*Ct*^* method (Pfaffl, 2001).

### Statistical Analysis

All statistical analyses were performed using the SPSS software (version 13.0; IBM, United States). Data were expressed as mean ± standard error of mean (SEM). Differences between two groups were analyzed using Student’s *t*-test. *p* < 0.05 was considered statistically significant.

## Results

### Protein Identification and Differential Expression Analysis

We used an untargeted LC-MS/MS approach to systematically investigate the effects of PEF on *E. coli* proteome. To this end, we applied PEF to freshly grown *E. coli* cells under abovementioned conditions that resulted in a 95 ± 2.0% reduction of bacterial population. In the meantime, we set up a control group in which cell aliquots from the same culture were similarly processed, but without the application of PEF. In total, we detected 16,829 peptides from our MS data and identified 2,252 proteins by sequence comparison with the Uniprot *E. coli* protein database. We then calculated the relative abundances of all identified proteins in the two experimental groups. Comparison of the expression data revealed 123 differentially expressed proteins based on the criteria of fold-change >1.5 or <0.67 and *p* < 0.05. Among them, 99 proteins exhibited significantly increased expression levels in PEF-processed *E. coli*, whereas 24 were found to be down-regulated (Table 1). The annotations, accession numbers and fold-change values of all differential proteins were summarized in Table 1. Notably, 33 proteins were confidently detected in at least two PEF-treated *E. coli* cultures but not in any of the controls, whereas another 19 were identified in at least two control samples but showed no apparent expression in the PEF treatment group. These candidates were therefore also considered as differentially expressed between the two groups and subsequently merged with the 123 proteins mentioned above. It is worth emphasizing that the reason that some proteins were only detected in two of the three samples in an experimental group could be caused by a combination of biochemical, analytical and statistical factors, such as miscleavage, ionization competition, ion suppression, peptide misidentification, ambiguous matching, etc. (Lazar et al., 2016).

**TABLE 1:** The significantly upregulated and downregulated proteins in *E. coli* after PEF-treated.

**Accession**	**Description**	**Coverage**	**Unique peptides**	**Folds Exe/Con**	***p*-value**
M9GK20	Citrate synthase, GN=ECMP0215612_0833	60.9	20	181.5173	0.0277
A0A0H3JNL0	Putative glucarate dehydratase, GN=ECs3648	5	1	28.3712	0.0232
V0UHA6	Octanoyltransferase, GN=lipB	35.1	3	18.3945	0.0002
W8T6L6	2-methylisocitrate lyase, GN=prpB	24	1	15.7546	0.0040
A0A2B7LUZ2	Aconitate hydratase B, GN=BMR23_08950	73.8	0	12.0020	0.0275
M9FZM4	2-methylcitrate dehydratase, GN=prpD	48.4	16	7.4411	0.02615
A0A0G3K113	Bifunctional protein PutA, GN=putA	49.8	1	5.9676	0.0224
W1G3P2	Aldehyde-alcohol dehydrogenase	53.6	1	5.8303	0.0452
W8ZMF2	Putative resistance protein, GN=yggT	10.1	2	4.0885	0.0211
A0A0K5XJK9	Ribosomal RNA small subunit methyltransferase F, GN=rsmF	10.1	2	3.6706	0.0003
A0A234Q1M9	GTP-binding protein, GN=AL530_011080	18.3	1	3.4667	0.0027
A0A234Y2B9	Putrescine-binding periplasmic protein, GN=RX35_02543	17.3	1	3.2918	0.0144
I0VQW1	2-dehydropantoate 2-reductase, GN=ECW26_29640	13.2	2	3.2735	0.0249
I4T327	Tryptophan permease, GN=EC54115_06619	9.6	3	3.2120	0.0008
A0A073V5W9	Dicarboxylate symporter family protein (Fragment), GN=AB08_2087	11.2	3	3.0766	0.0312
Q8 × 646	Uncharacterized protein, GN=ECs2348	56.1	3	3.0709	0.0023
A0A2T7Y366	FeS assembly scaffold SufA, GN=sufA	16.7	1	2.9581	0.0116
A0A229AFZ0	Lactam utilization protein LamB, GN=CDL37_20835	61.4	1	2.8832	0.0014
W9AMD8	Phosphate-binding protein PstS, GN=pstS	50.3	5	2.8444	0.0023
W1FRP3	ATP-dependent Clp protease ATP-binding subunit ClpA	34.4	0	2.7513	0.0314
W1FTY7	Galactose/methyl galactoside ABC transport system, D-galactose-binding periplasmic protein MglB (TC 3.A.1.2.3)	49.4	1	2.6992	0.0013
W1F3 × 3	Phosphate transport ATP-binding protein PstB (TC 3.A.1.7.1)	24.7	5	2.6913	0.0155
A0A2H9B301	L-cystinetransporter, GN=CG691_13490	8.7	2	2.5515	0.0080
A0A193RPM2	Ornithine decarboxylase, GN=WM48_15995	11.3	6	2.4748	0.0009
A0A0T5XPP6	Molybdopterin biosynthesis protein MoeA, GN=AOX65_16030	3.2	1	2.4707	0.0008
A0A2T8H312	Bifunctional glutamine amidotransferase/anthranilate phosphoribosyltransferase, GN=APX76_17630	35.3	11	2.4593	0.0006
W8SPU9	Nicotinate phosphoribosyltransferase, GN=pncB	21.2	6	2.3065	0.0056
A0A1 × 3L920	Protein AmpE, GN=EAXG_02432	14.8	1	2.2675	0.0084
D7 × 9F1	Uncharacterized protein (Fragment), GN=HMPREF9552_03278	50	2	2.2191	0.0032
W9ADK0	Uncharacterized protein, GN=ycbJ	20.2	4	2.1980	0.0033
A0A0T5XIP1	Regulator of ribonuclease activity A, GN=rraA	27.3	1	2.1974	0.0017
W9ADR5	Uncharacterized protein, GN=yccJ	60	3	2.1729	0.0246
W1WSH0	Protein mioC, GN=Q609_ECAC01910G0002	44.9	3	2.1698	0.0108
A0A1 × 3KHH3	Alcohol dehydrogenase YqhD, GN=EATG_01859	39	1	2.1604	0.0003
W8ZNL1	UPF0250 protein YbeD, GN=ybeD	55.2	4	2.1440	0.0009
A0A096ZJW9	Sigma-38 (Fragment), GN=rpoS	38.7	9	2.1201	0.0050
V8FHS8	Spermidine/putrescine ABC transporter substrate-binding protein, GN=Q458_15770	56.2	15	2.0609	0.0268
A0A2B7MPJ0	Dihydroxyacetone kinase subunit DhaM, GN=BMR23_01885	17.4	1	2.0210	0.0283
W1 × 9K1	Galactonate operon transcriptional repressor (Fragment), GN=Q609_ECAC00550G0001	35	4	2.0176	0.0017
W8ZNL8	Nuclease SbcCD subunit C, GN=sbcC	5.8	4	2.0107	0.0127
W8ZJY8	Uncharacterized protein, GN=yecA	35.7	5	2.0036	0.0011
W8ZVZ2	Sulfate adenylyltransferase subunit 1, GN=cysN	32.2	12	2.0004	0.0294
W9AED4	Fatty acid metabolism regulator protein, GN=fadR	56.9	10	1.9864	0.0041
W8STE3	BolA DNA-binding transcriptional dual regulator, GN=bola	42.3	3	1.9619	0.0064
V6FYL6	Bifunctional polymyxin resistance protein ArnA, GN=arnA	25.9	13	1.9311	0.0075
A0A0K9TED1	Smgprotein, GN=ERYG_01892	45.3	3	1.8818	0.0119
W8ZV88	Sulfite reductase [NADPH] hemoprotein beta-component, GN=cysI	30.5	6	1.8737	0.0141
W1WPC4	Cell division protein ZapB, GN=zapB	88.6	7	1.8712	0.0026
W8T248	Protein YciE, GN=yciE	51.8	6	1.8429	0.0174
W1HGY9	6,7-dimethyl-8-ribityllumazine synthase, GN=ribH	87.2	8	1.8411	0.0024
A0A2S7HHN4	Uncharacterized protein (Fragment), GN=C5P43_35025	34.2	3	1.8367	0.0432
M9GGR7	Protein rof, GN=rof	63.3	3	1.8317	0.0361
W1F1D7	Anthranilate synthase component 1	32.5	14	1.8300	0.0208
V8KFY8	Protein CsiD, GN=csiD	64.6	17	1.8290	0.0061
W8ZNY2	Transcriptional regulator ModE, GN=modE	51.9	8	1.8284	0.0121
D6I850	YciFprotein, GN=ECDG_01154	45.2	6	1.8177	0.0022
V0YAU0	Uncharacterized protein (Fragment), GN=HMPREF1608_01019	24.2	1	1.8173	0.0434
A0A0K4H5N9	Transcriptional regulator, GN=ERS085411_00773	27.4	10	1.7957	0.0022
V0YHQ2	DNA recombination protein RmuC, GN=HMPREF1608_02578	20.6	8	1.7851	0.0374
A0A1 × 1LNA3	Formate dehydrogenase-N subunit alpha, GN=fdnG	41.6	30	1.7653	0.0156
V6FUE1	Pimeloyl-[acyl-carrier protein] methyl ester esterase, GN=bioH	30.1	4	1.7541	0.0492
W8U272	Fused mannitol-specific PTS enzymes: IIA components/IIB components/IIC components, GN=mtlA	36.1	14	1.7486	0.0103
A0A1 × 3KKE8	Transketolase, GN=EATG_02582	37.5	1	1.7098	0.0264
W8ZZB2	Regulator of ribonuclease activity A, GN=menG	50.3	4	1.7003	0.0006
W8ZPR2	Protein TusB, GN=yheL	12.6	1	1.6993	0.0332
S0VE10	PTS system trehalose-specific EIIBC component, GN=WE7_05383	18.4	6	1.6949	0.0410
W8TSM7	C-lysozyme inhibitor, GN=ivy	46.6	5	1.6947	0.0206
W9AP24	Regulator of ribonuclease activity B, GN=yjgD	39.1	3	1.6871	0.0056
W8ZR12	Tryptophan synthase alpha chain, GN=trpA	59.7	1	1.6803	0.0021
W1EYP8	L-proline glycine betaine ABC transport system permease protein ProW (TC 3.A.1.12.1)	16.2	2	1.6793	0.0206
W8U2G6	Carboxymethylenebutenolidase, GN=ysgA	38	5	1.6667	0.0317
W8ZZG4	Regulator of sigma D, GN=yjaE	27.8	3	1.6666	0.0034
A0A0T5XE89	Threonine synthase, GN=AOX65_13005	13.1	1	1.6576	0.0007
T9DJA4	Glutaminase, GN=glsA	48.4	3	1.6534	0.0071
W1 × 6R0	Phosphate starvation-inducible protein psiF, GN=Q609_ECAC01403G0002	39.6	4	1.6472	0.0342
W8ZRC1	Uncharacterized protein, GN=EC958_1708	73	12	1.6406	0.0150
A0A2H9CSQ5	Deoxyuridine 5-triphosphate nucleotidohydrolase, GN=coaBC	45.9	15	1.6347	0.0003
W1EQP2	Tryptophan synthase beta chain, GN=trpB	38.8	2	1.6319	0.0017
A0A0J2EWU6	Dihydroorotate dehydrogenase (quinone), GN=pyrD	36	9	1.6316	0.0007
W1EWD5	Transcriptional regulator YcjW, LacI family, possibly involved in maltodextrin utilization pathway	15.9	1	1.6275	0.0138
W9AB69	Uridylatekinase, GN=pyrH	45.2	7	1.6140	0.0320
W8ZNI9	Regulator of nucleoside diphosphate kinase, GN=rnk	26.5	2	1.6051	0.0140
S1J5S6	Protein AroM, GN=A1WS_00874	16	1	1.6048	0.0490
W1XC14	Iron-sulfur cluster insertion protein ErpA, GN=erpA	22.8	2	1.5893	0.0095
U9YVN3	D-methionine-binding lipoprotein MetQ, GN=HMPREF1599_05823	20.2	2	1.5838	0.0117
W1VVN8	Replicative DNA helicase, GN=Q609_ECAC02906G0004	25.3	8	1.5828	0.0111
A0A2A3VGN6	UDP-4-amino-4-deoxy-L-arabinose – oxoglutarate aminotransferase, GN=arnB	20.3	2	1.5763	0.0295
H4UMP9	Inner membrane protein ypjD, GN=ypjD	8.2	1	1.5698	0.0065
W1F6U7	Chromosome partition protein MukE, GN=mukE	37.3	6	1.5425	0.0203
W8SQ29	GST-like protein with glutathione S-transferase domain protein YliJ, GN=gstB	52.4	8	1.5391	0.0072
A0A2I6JDE7	Arabinose ABC transporter substrate-binding protein, GN=CRT55_12445	32	2	1.5267	0.0465
W8SS44	Hydrolase, GN=ycaC	48.6	3	1.5255	0.0104
W1EY44	Zinc transport protein ZntB	13.4	3	1.5160	0.0148
W8ZUR8	Uncharacterized protein, GN=elaB	44.6	4	1.5169	0.0186
H4LI45	Peptidyl-prolyl *cis*-*trans* isomerase, GN=slyD	68	6	1.5151	0.0356
W1WTW0	Arginine repressor, GN=argR	42.9	4	1.5101	0.0013
V2T0T4	ABC transporter periplasmic-binding protein, GN=G723_01103	17.6	5	1.5076	0.0307
A0A2R9W5R2	Primosomal protein DnaT (Fragment), GN=C1I57_22015	33.6	3	1.5058	0.0426
A0A2R9W0 × 1	L-arabinose isomerase (Fragment), GN=C1I57_30235	10.6	3	1.5021	0.0302
W8ZM76	Uncharacterized protein, GN=yadG	19.2	7	0.6651	0.0173
W8ZVN7	Putative YhbH sigma 54 modulator, GN=EC958_2900	59.3	6	0.6611	0.0349
M9FWG9	Arginine transport ATP-binding protein ArtP, GN=artP	23.1	4	0.6609	0.0215
W9ACD0	Proofreading thioesterase EntH, GN=ybdB	18.2	2	0.6451	0.0007
A0A0A0FBI3	Uncharacterized protein, GN=EL76_3316	16	1	0.6406	0.0041
W8ZQK0	Protoporphyrinogen oxidase, GN=hemG	30.4	5	0.6399	0.0391
T6MLM8	DNA polymerase, GN=G749_00151	0.9	1	0.6373	0.0079
W1W6N1	1,4-dihydroxy-2-naphthoate octaprenyltransferase, GN=menA	6.5	2	0.6348	0.0084
E5FGE1	WeiT, GN=weiT	21.8	7	0.6195	0.0005
W9AKV1	Uncharacterized protein, GN=yrdD	13.3	2	0.6191	0.0053
W9AHV7	Anaerobic glycerol-3-phosphate dehydrogenase subunit C, GN=glpC	18.4	7	0.6148	0.0308
I0VVR9	Nitrite reductase (NAD(P)H), large subunit, GN=ECW26_12260	10.9	9	0.6079	0.0023
A0A2S7H9V9	Phosphate acyltransferase PlsX (Fragment), GN=plsX	10.1	2	0.6067	0.0152
W1WWC3	Outer membrane lipoprotein Blc, GN=Q609_ECAC01725G0007	11.5	2	0.6030	0.0401
G0F3L2	Polyketide cyclase/dehydrase and lipid transport family protein, GN=UMNF18_3418	42.6	5	0.6011	0.0032
V0SC60	Uncharacterized protein, GN=HMPREF1595_04697	43.4	7	0.6010	0.0207
W9AKX1	50S ribosomal protein L30, GN=rpmD	57.6	4	0.6008	0.0219
W1F1V3	CDP-diacylglycerol pyrophosphatase, GN=cdh	25.1	6	0.5951	0.0127
W8ZP35	Uncharacterized protein, N=yqjE	20.9	4	0.5847	0.0081
W1WR46	Inner membrane protein ylaC, GN=Q609_ECAC01779G0002	43.6	6	0.5658	0.0010
A7ZI09	Uncharacterized protein, GN=EcE24377A_0284	11	2	0.5396	0.0048
W1WXG0	Uncharacterized protein, GN=Q609_ECAC01601G0009	52.7	5	0.5229	0.0006
W8ZSI0	Cyclopropane fatty acyl phospholipid synthase, GN=EC958_1883	34.6	11	0.4375	0.0019
A0A1V2T523	DeoR family transcriptional regulator (Fragment), GN=BET08_18455	7.9	2	0.1134	0.0018

**FIGURE 2:**
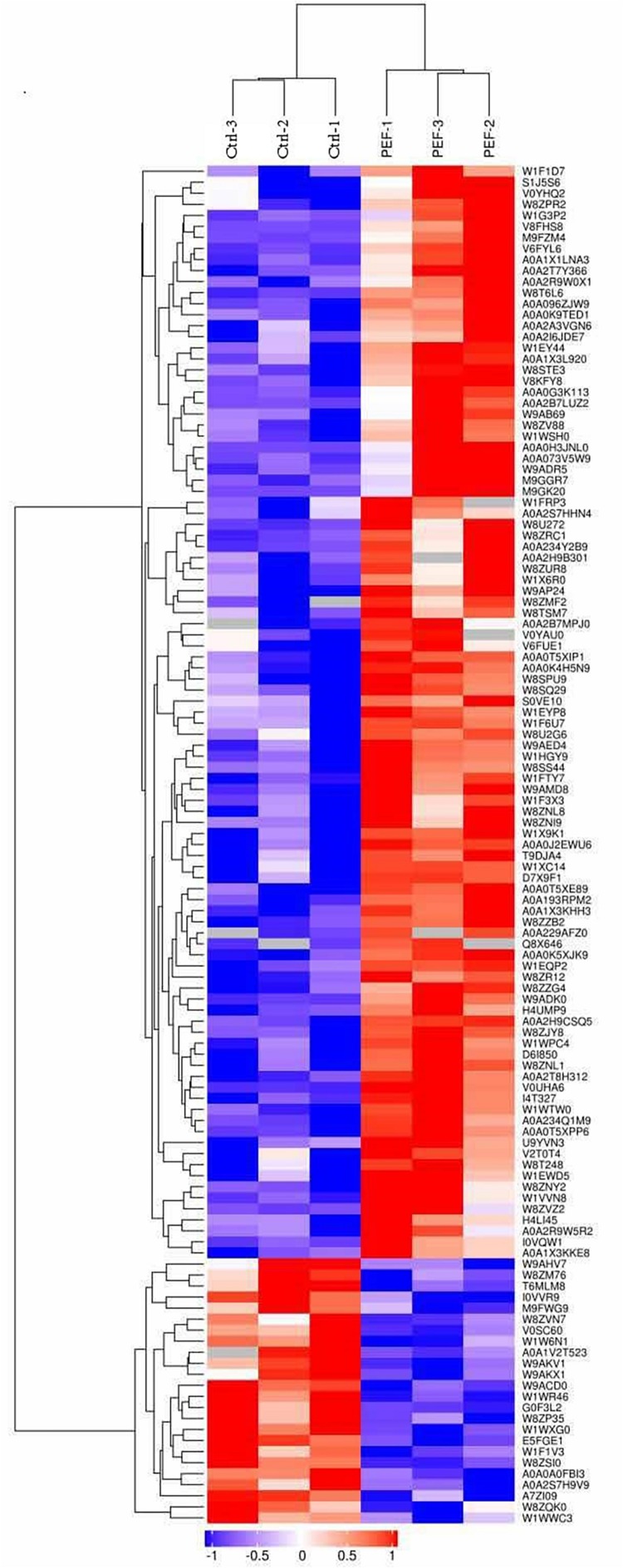
Hierarchical clustering of 123 differentially expressed proteins (confidently detected in all six samples; fold-change >1.5 or <0.67, and *p* < 0.05; see Table 1). Results are illustrated using a heat map with a dendrogram. The Uniprot ID of each protein is listed in the column to the right of the heat map. The color bar located below the heat map denotes the extent of change in expression level, with red indicating up-regulation and blue down-regulation.

To better compare the global proteomic profiles of the PEF-treated *E. coli* samples and the controls, we performed hierarchical clustering analysis of the 123 differentially expressed proteins and illustrated the results in an expression heat map with dendrogram (Figure 2). As depicted, the plot indicated clear differences in the number of detected proteins between the two experimental groups.

### Functional Analysis of the Differentially Expressed Proteins

**FIGURE 3:**
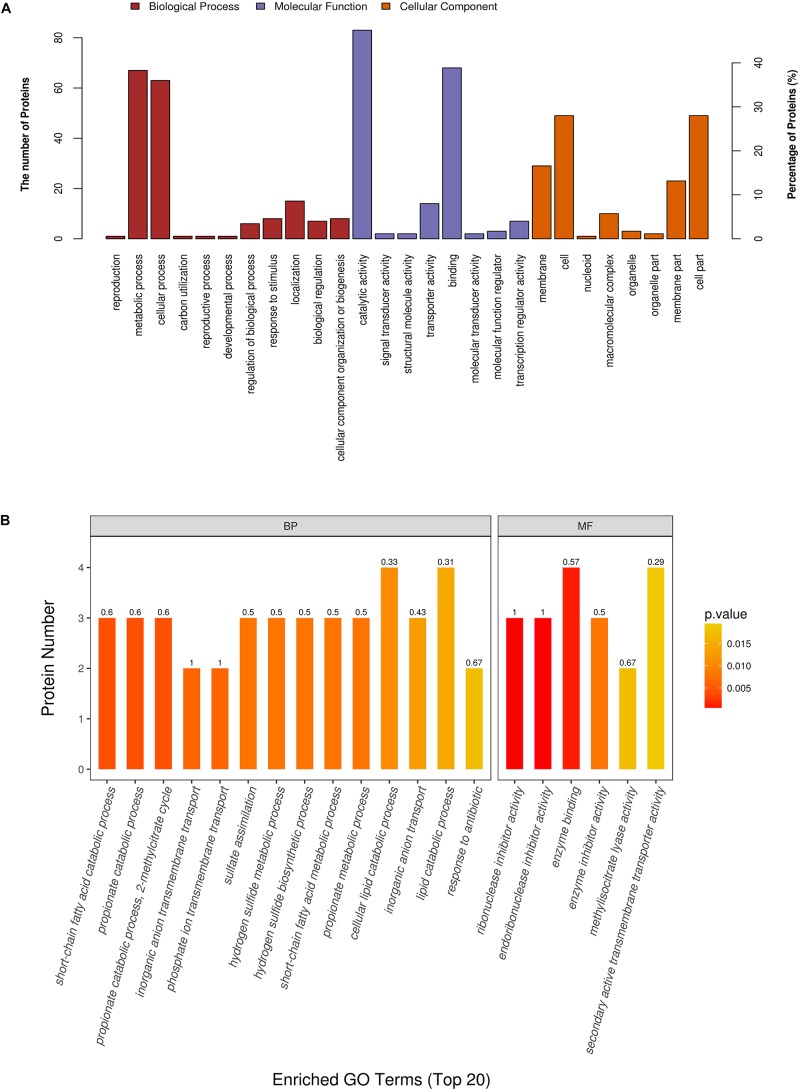
Gene ontology (GO) annotation and enrichment analysis of all 175 differentially expressed proteins. **(A)** The primary *Y* axis denotes the number of annotated proteins categorized to each GO term. The secondary *Y* axis represents the percentage of annotated proteins belonging to each GO term in all differential proteins. GO terms are classified into three subcategories, including biological process (BP, red), molecular function (MF, purple) and, cellular compartment (CC, orange). **(B)** The color gradient from orange to red represents the *p*-value; the closer the color to red, the lower the *p*-value and the higher the significance level corresponding to the enrichment. The small number above each column is the rich factor, which denotes the ratio of the number of differential proteins enriched to a given GO term to the number of all annotated proteins categorized to the same GO term.

We next performed functional analysis of all 175 differentially expressed proteins (including the ones that could not be confidently detected in all samples as explained above) to shed light on their potential molecular and cellular roles. GO annotation by Blast2GO and InterProScan indicated that the differential proteins were predominantly involved in catalytic activity (47.43%) and binding (38.86%) under the subcategory of molecular function (Figure 3A). For biological process, the overwhelming majority of the candidates could be assigned to GO terms of cellular process (36%) and metabolic process (38.29%) (Figure 3A). Subsequent GO enrichment analysis demonstrated that PEF-treated *E. coli* underwent a wide range of metabolic alterations, such as those associated with sulfate, sulfide, propionate, 2-methylcitrate and short-chain fatty acids. In addition, we also identified significant changes in the transmembrane transport of inorganic anions, particularly phosphate ions. Other notably enriched GO terms included enzyme binding and enzyme inhibitor activity, including ribonuclease inhibitor activity, endoribonuclease inhibitor activity and methyl isocitrate lyase activity (Figure 3B).

**FIGURE 4:**
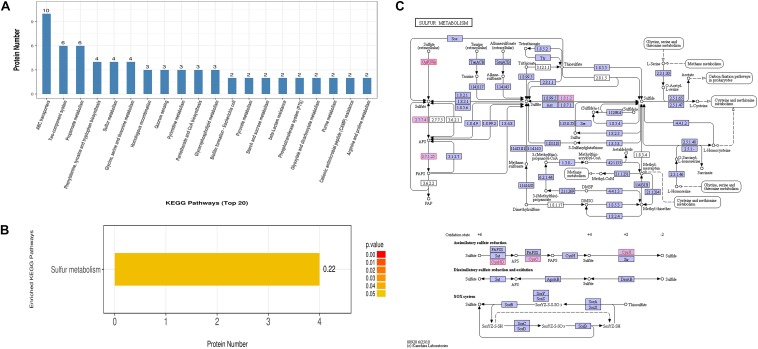
KEGG pathway and enrichment analysis of all 175 differentially expressed proteins. **(A)** The *Y* axis indicates the number of annotated proteins that are categorized into each KEGG pathway. Only the top 20 pathways are shown. **(B)** Sulfur metabolism is the only KEGG pathway found to be significantly enriched. **(C)** An illustration of the cellular processes associated with sulfur metabolism pathways. All differential proteins captured in this study are highlighted in red. Small circles (o) represent small-molecule metabolites (Source: Kanehisa et al., 2019).

On the other hand, the differentially expressed proteins could be mapped to 81 KEGG pathways. The top 5 KEGG pathways with the greatest numbers of protein candidates included those associated with ABC transporters, two-component system, propanoate metabolism, phenylalanine, tyrosine and tryptophan biosynthesis, as well as sulfur metabolism (Figure 4A). Further examination suggested that sulfur metabolism was significantly enriched (Figure 4B), which comprised four differentially expressed proteins W8ZVZ2 (sulfate adenylyltransferase subunit 1, EC:2.7.7.4, CysND), W8ZV88 (Sulfite reductase [NADPH] hemoprotein beta-component, EC:1.8.1.2, CysJI), I0VXI2 (Sulfate-binding protein, CysPUWA) and A0A1V2T430 [Adenylyl-sulfate kinase (Fragment), EC:2.7.1.25, CysC]. Among them, W8ZVZ2 and W8ZV88 were up-regulated in PEF-treated *E. coli* cells by 2.0-fold and 1.8-fold, respectively, compared to the untreated cells, whereas I0VXI2 and A0A1V2T430 were only detected in PEF-treated cells. The metabolic roles of these proteins were illustrated in Figure 4C.

### PPI Analysis

**FIGURE 5:**
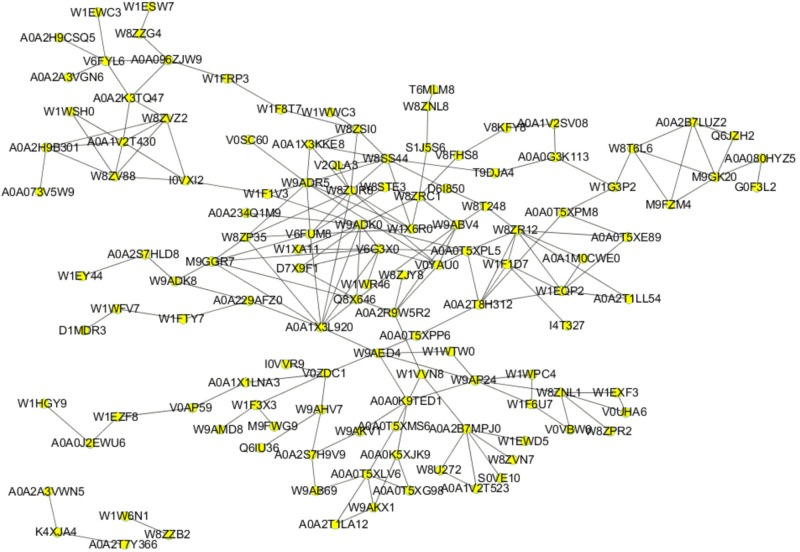
PPI network based on 124 proteins from all 175 differentially expressed proteins. Nodes and lines represent the protein and their interactions, respectively.

It is a well-established concept that cellular processes are often the results of specific interactions between two or more proteins rather than the actions of a single protein. Based on these considerations, we constructed a PPI network of all the differentially expressed proteins that we identified in order to gain a deeper understanding of how the various PEF-altered biological functions in *E. coli* cells were interconnected with each other. As shown in Figure 5, the network map comprised 123 nodes and 208 interactions. W8ZUR8 (uncharacterized protein elaB), predicted by GO annotation to participate in ribosome binding, represented the largest node with ten putative associations. Other differential proteins predicted to interact with seven or more partners included W9ADR5 (uncharacterized protein yccJ), W9ADK0 (uncharacterized protein cbJ), A0A1 × 3L920 (beta-lactamase induction protein AmpE), W8SS44 (hydrolase ycaC), W8ZR12 (tryptophan synthase alpha chain, trpA), and V6FUM8 (universal stress protein B UspB). Notably, all these proteins were up-regulated in the PEF treatment group, with V6FUM8 detected only in PEF-processed samples.

### Data Verification by PRM Analysis

The proteomic data were verified by selecting five differentially expressed proteins for PRM analysis, including M9GK20 (Citrate synthase), W8ZVZ2 (Sulfate adenylyltransferase subunit 1), V6FYL6 (Bifunctional polymyxin resistance protein ArnA), M9FZM4 (2-methylcitrate dehydratase) and W9AMD8 (Phosphate-binding protein PstS). We quantified the levels of 1–3 unique peptides for each selected protein in the PEF-processed *E. coli* and untreated controls. As shown in (Table 2), all five proteins were significantly up-regulated as a result of the PEF treatment, which was consistent with the results of the untargeted proteomic analysis. This suggested that the proteomic data that we obtained were sufficiently reliable for subsequent interpretation.

**TABLE 2:** Comparison of quantification results between label-free and PRM.

**Accession**	**Description**	**PRM results**	**Label-free results**
		**Ratio _PEF/Ctrl**	**Ratio _PEF/Ctrl**
M9GK20	Citrate synthase	6.96 (up)	181.5172 (up)
W9AMD8	Phosphate-binding protein	3.60 (up)	2.8444 (up)
M9FZM4	2-methylcitrate dehydratase	7.32 (up)	7.4411 (up)
V6FYL6	Bifunctional polymyxin resistance protein ArnA	1.55 (up)	1.9311 (up)
W8ZVZ2	Sulfate adenylyltransferase subunit 1	1.87 (up)	2.0004 (up)

In the present study, we systematically analyzed PEF-induced changes in the protein expression profile of *E. coli* through MS-based label-free quantitative proteomics. Our experimental data echoed previous findings (Rivas et al., 2013; Yun et al., 2016; Zhao et al., 2018), that exposure to PEF exerted significant detrimental effects on cell membrane integrity, triggered a host of cellular stress response mechanisms, and altered important metabolic pathways. The results of our current study could increase our understanding of the mechanisms responsible for PEF-induced inactivation of bacterial cells in food products.

### qRT-PCR Validation

**FIGURE 6:**
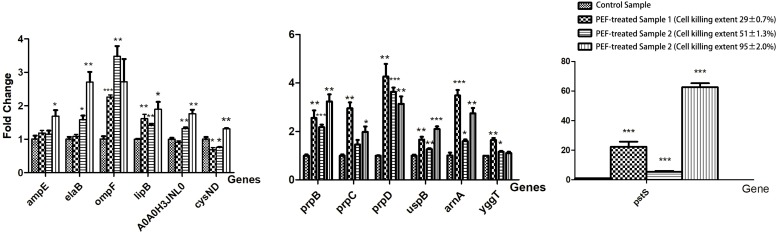
The effect of PEF treatment on the expression of selected *E. coli* genes under different pulse conditions (^∗^*p* < 0.05, ^∗∗^*p* < 0.01, ^∗∗∗^*p* < 0.001). The gene expression data are arranged in separate column charts due the fold-change values of pstS being significantly greater than those of other genes. The pulse conditions and cell killing extent for each sample are as follows: control sample: untreated; PEF-treated sample 1: pulse intensity – 2.88 kV cm^–1^, pulse number – 62, pulse duration – 82 μs, cell killing extent – 29 ± 0.7%; PEF-treated sample 2: pulse intensity – 6.10 kV cm^–1^, pulse number – 54, pulse duration – 77 μs, cell killing extent – 51 ± 1.3%; PEF-treated sample 3: pulse intensity – 14.5 kV cm^–1^, pulse number – 26, pulse duration – 67 μs, cell killing extent – 95 ± 2.0%.

We selected a list of significantly up-regulated proteins and measured their mRNA levels at different PEF intensities (which resulted in different cell killing levels). The detailed information and the selection rationale for each protein were summarized in (Table 3). As illustrated in Figure 6, all candidates demonstrated increased transcription at all PEF intensities that we tested, confirming the validity of our proteomic results. However, the genes showed different expression trends with increasing PEF intensities. In most cases, we did not observe a positive correlation between the mRNA level of the gene and the level of cell death. These results suggested that PEF treatment could directly stimulate the expression of the *E. coli* proteins, though indirect contribution via cell lysis could not be completely eliminated. In addition, at high intensities, PEF exerted a clear inhibitory effect on the transcription of most of the genes.

**TABLE 3:** Differential protein use for fluorescence quantification PCR.

**Protein**	**Gene Name**	**Reason for verification**
M9GK20	prpC	Transferase activity, transferring acyl groups, acyl groups converted into alkyl on transfer, tricarboxylic acid cycle
W9AMD8	pstS	Part of the ABC transporter complex PstSACB involved in phosphate import
M9FZM4	prpD	Propionate catabolic process, 2-methylcitrate cycle
W8ZMF2	yggT	Resistance protein
ECs3648	A0A0H3JNL0	Amino acid metabolism
V0UHA6	lipB	Catalyzes the transfer of endogenously produced octanoic acid from octanoyl-acyl-carrier-protein onto the lipoyl domains of lipoate-dependent enzymes.
W8T6L6	prpB	Catalyzes the thermodynamically favored C-C bond cleavage of (2R,3S)-2-methylisocitrate to yield pyruvate and succinate.
W8ZVZ2	cysND	Sulfur metabolism
V6FYL6	arnA	The modified arabinose is attached to lipid A and is required for resistance to polymyxin and cationic antimicrobial peptides.
V6FUM8	uspB	Universal stress protein
W8ZUR8	elaB	Ribosome binding
A0A1 × 3L920	AmpE	PPI network important node
W9ADK8	ompF	Outer membrane protein

## Discussion

Notable among the most up-regulated proteins in the PEF treatment group are several enzymes related to the methylcitrate cycle and the TCA cycle, including 2-methylisocitrate lyase (PrpB; W8T6L6), the dual-functional aconitate hydratase B (alsoaconitase, AcnB; A0A2B7LUZ2), 2-methylcitrate dehydratase (PrpD; M9FZM4), and the rate-limiting citrate synthase (prpC; M9GK20). The methylcitrate cycle functions in a similar manner as the TCA cycle, and is responsible for converting propionyl-CoA to pyruvate for further catabolism (Luo et al., 2016; Serafini et al., 2019). As a result, both the TCA cycle and the methylcitrate cycle are functionally connected to and downstream of the β-oxidation of fatty acids. Since increased lipid oxidation is a well-established phenomenon under oxidative stress (Esterbauer et al., 1991) or PEF treatment (La et al., 2015; Yun et al., 2016), we speculate that the augmented expression of TCA or methylcitrate cycle-associated enzymes could reflect a pre-emptive metabolic defense mechanism of the stressed *E. coli* cells. This is supported by the results discussed in several studies with regard to the anti-oxidative roles of the TCA cycle (Tretter and Adam, 2000; Mailloux et al., 2010). It is worth mentioning that AcnB contains a critical Fe-S cluster that is particularly susceptible to oxidative stress. In fact, there is evidence that excessive accumulation of reactive oxygen species (ROS) could dramatically reduce the protein level of AcnB in superoxide dismutase-deficient *Salmonella enterica* (Thorgersen and Downs, 2009). Therefore, the effects of PEF treatment and oxidative stress on the expression of AcnB and other enzymes mentioned above could be both stimulatory and inhibitory. Another top-ranking up-regulated enzyme is bifunctional proline utilization A (PutA; A0A0G3K113), which catalyzes the conversion of proline to glutamate via two consecutive steps of dehydrogenation (Menzel and Roth, 1981). PutA is known to be involved in redox homeostasis and its deletion in *E. coli* has been shown to substantially increased cell susceptibility to oxidative injuries (Zhang et al., 2015). It has been postulated that the protective role of PutA against oxidative stress could be attributed to the generation of a low level of hydrogen peroxide by proline oxidative metabolism, which builds resistance in cells in a pre-adaptive manner (Zhang et al., 2015). Taken together, these results suggested that PEF treatment and the possibly associated oxidative stress could activate the stress response mechanism in the *E. coli* cells, leading to induced expression of various oxidation-combating enzymes.

Cell membrane functions as the main barrier against harmful substances and regulates the exchange of essential materials between the cytoplasm and the environment. When exposed to a strong electric field, a cell becomes polarized due to the migration of ions in its cytoplasmic fluid. The realignment of opposite charges on both sides of the cell creates electrostatic attraction that squeezes the cell and its membrane (Zimmermann et al., 1974). Meanwhile, the various membrane constituents of the cell are also perturbed by the electromechanical forces. The polar lipids, including phospholipids and cholesterol, represent obvious targets. As the electric field intensifies, both electromechanical compression and polar lipid rearrangement will reach a breaking point that leads to pore formation (Zimmermann et al., 1974; Serpersu et al., 1985; Weaver and Chizmadzhev, 1996; Unal et al., 2002; Liu Z.W. et al., 2016). One of the direct consequences of this is increased membrane permeability, which not only allows entry of deleterious substances, but also generates an osmotic imbalance (Aronsson et al., 2005). Obviously, the electric induction of a few small pores is usually non-lethal, as the fluidity of the biolipid layer allows rapid restoration of a stable membrane structure (Aronsson et al., 2005). However, a sufficiently strong electric field is capable of creating an increased number of large pores on the membrane, leading to irreversible cell damage, disruption, and eventually death (Locke et al., 2006; Lin et al., 2012). Numerous studies have been performed to determine the boundary between PEF-induced temporary electroporation and permanent sterilization. Based on these results, it is generally believed that irreversible cell injury occurs when the field strength reaches 5–15 kV⋅cm^–1^ (Guerrero and Welti, 2016). This is consistent with observations that PEF at an intensity above 15 kV⋅cm^–1^ is necessary for membrane breakdown (Lampe, 1999; Lebovka et al., 2001). However, some researchers have noticed microbial resistance to field strengths as high as 19 kV⋅cm^–1^ (Ulmer et al., 2002). As a result, it has been argued that PEF with a field strength of 25 kV⋅cm^–1^ or above should be used to ensure pathogen inactivation in food processing (Toepfl et al., 2007).

Overall, 29 of the 175 differentially expressed proteins that we identified in our current proteomic study are localized on the cell membrane and/or potentially involved in membrane functions. Particularly, 16 of these proteins participate in the transmembrane transport of various small metabolites and macromolecules, including phosphate ions, zinc, glycine betaine, trehalose, as well as short peptides, carbohydrates and lipids in general. There is ample evidence that sugar molecules, especially trehalose, exert a protective effect on the physical structure and integrity of cell membrane (Crowe, 2002). It has been hypothesized that carbohydrates can increase the internal cohesion of cell membrane by forming stabilizing hydrogen bonds with its various components (Locke et al., 2006). Another theory argues that sucrose and trehalose can mitigate mechanical disruption of cell membrane by forming an amorphous glass structure (Crowe et al., 1998). In support of these roles, Pereira et al. demonstrated that trehalose could effectively reduce the physical stress that stretching induced on cell membrane by forming hydrogen bonds with the polar lipid components (Pereira and Hünenberger, 2008). Glycine betaine is another well-known cryoprotectant and osmoprotectant that is often accumulated in cells in response to external abiotic stress stimuli. Several studies have shown that transport of glycine betaine across bacterial cell membrane can be activated by osmotic shock and low temperature (Gerhardt et al., 2000; Guillot et al., 2000). Therefore, it seems more plausible that the altered expression of membrane transporters could be a cellular defense mechanism against PEF-induced structural disruptions, though it is also possible that the electromechanical forces might directly affect the expression or stability of these proteins.

It is worth mentioning that some of the differential membrane proteins were only detectable in the PEF treatment group. A careful survey of their functions implied that these proteins might have been activated by PEF for different reasons. The outer membrane protein F (ompF; W9ADK8) directly contributes to pore formation and has been shown to help *E. coli* cells maintain their structural stability (Nogami and Mizushima, 1983). However, the effects of ompF seem to be mixed, as Tan et al. (2017) reported that its deletion led to substantial enhancement of membrane integrity. Based on these findings, we speculated that the augmented expression of ompF could either be a sign of increased membrane damage or reflect the cellular response to PEF-induced perturbations of intracellular compounds. Universal stress protein B (uspB; V6FUM8) and other members of the USP family can be induced by a wide range of abiotic stress stimuli (Nachin et al., 2005) and participate in a variety of cytoprotective activities such as DNA protection (Gustavsson et al., 2002), arrest of cell growth (Neidhardt and Nyström, 2010) and re-adaptation of cell metabolism to nutrient shortage (Tkaczuk et al., 2013). The *E. coli* sensor histidine kinase (A0A2R9W207) belongs to the two-component signal-transducing system implicated in stimuli perception (Mascher et al., 2006). Thus, its stimulated expression could be the result of increased PEF-dependent stresses. The prenyltransferase family protein (ubiA; A0A080HYZ5) is responsible for lipidating a wide range of suitable acceptor compounds. Recent studies pointed out that many lipophilic products of prenyltransferase serve as important components of bacterial cell membrane (Li, 2016), raising the possibility that its up-regulation by PEF could be a cellular repair mechanism. We are especially intrigued to discover that a macrolide-specific efflux protein (MacA; H4L9U9) exhibited detectable expression only in PEF-treated cells. Mostly known for their antibiotic activities, macrolides are recently speculated to play an additional role in membrane organization. For example, Tyteca et al. (2003) examined the mechanism of azithromycin-based inhibition of endocytosis and found that the macrolide compound could diminish membrane fluidity by binding to the polar heads of phospholipids. This is further corroborated by findings that macrolide efflux pumps can be up-regulated by stress signals unrelated to drug resistance (Bolla, 2014).

In addition to Rivas et al.’s (2013) protein profiling study based on 2D gel electrophoresis, there have been several other proteomics or transcriptomics investigations of PEF-treated *E. coli*. Chueca and coworkers treated *E. coli* MG1655 cells, buffered at pH 4.0, with 50 exponential waveform electric pulses with a frequency of 0.08 Hz and pulse duration of 2 μs, followed by DNA microarray-based transcriptomic analysis (Chueca et al., 2015). Based on their data, a total of 47 genes showed differential expression as a result of the treatment. One of their major findings was the significantly elevated mRNA expression of several genes associated with the TCA cycle, including (*sdhA*, *sdhB*, *sdhC*, *sdhD*). Although these genes were different from those that encoded the differential proteins identified in this study, their finding of a link between PEF treatment and alterations in TCA-associated cell metabolism mirrored what our results unearthed in this study. In addition, Chueca et al.’s (2015) study also indicated the up-regulation of genes involved in cell respiration and membrane function, as well as the down-regulation of several mRNAs related to acid shock response, possibly as a result of the mild acidic environment of the cell suspension during the treatment. On the other hand, it has come to our attention that a recent PEF study by Guionet et al. (2014) did not result in as extensive changes in cells as what we have observed in our current study. Specifically, they reported that treating *E. coli* cells with 500 consecutive 60-ns pulses at 10^7^ V m^–1^, followed by a 1-h proteomic recovery in LB at room temperature, only led to the differential expression of one protein (Guionet et al., 2014). We speculated that the discrepancies could have stemmed from the fact that they used considerably shorter pulses and detected proteins by two-dimensional gel electrophoresis, which, in theory, tends to afford a lower diversity of protein candidates.

The development of resistance in pathogenic microbes against PEF treatment poses a significant problem to food preservation. In some cases, such resistance seems to be temperature- and/or pH-dependent (Somolinos et al., 2008), suggesting the involvement of enzymes and/or other bioactive molecules that confer damage mitigation. Liu and coworkers pointed out that the composition of membrane fatty acids plays a key role in the PEF resistance of *E. coli* by regulating membrane fluidity (Liu et al., 2017). A similar conclusion has also been obtained by Cebrián (Cebrián et al., 2016). On the other hand, microbial cells can activate an oxidative stress response upon PEF treatment, which has been shown to produce excessive ROS (Pakhomova et al., 2012). As evidence, Tanino et al. (2012) reported the up-regulation of several genes associated with oxidation stress, including those encoding glutathione synthase and superoxide dismutase, in PEF-stressed yeasts. Furthermore, the authors confirmed that the attenuation of such oxidative injuries strongly correlated with the PEF resistance of the cells (Tanino et al., 2012). García et al. (2010) suggested that enzymes involved in lipid biosynthesis and energy production are required for post-PEF membrane repair. In support of these findings, we demonstrated that PEF treatment significantly augmented the expression of several proteins involved in sulfate transport and reduction. This would in turn result in increased levels of sulfide and other reductive sulfur-containing species, presumably exerting a cytoprotective effect against PEF-induced oxidative stress (Shatalin et al., 2011; Ono et al., 2014; Wu and Gao, 2017). Taken together, these studies offered clear evidence that microorganisms can adopt a number of strategies to combat the detrimental effects of PEF.

In summary, we reported the first systematic proteomic profiling study on PEF-treated *E. coli*. The results that we obtained suggested that the application of PEF could significantly impact the membrane integrity of the cells and could potentially stimulate a wide range of cellular defense mechanisms. These results can help researchers better understand PEF-induced microbial inactivation and develop more effective PEF-based food processing methods.

## Data Availability Statement

The datasets generated for this study can be found in the ProteomeXchange Consortium using accession number PXD014365 and also via the iProX partner repository (Ma et al., 2019).

## Author Contributions

ZL, LZ, and YS completed the experimental design and modification of the manuscript. ZL, QZ, NH, and XS *E. coli* completed the experimental part. LL, LJ, and YL completed the writing and translation of the manuscript. YP, XS, and YS completed the data analysis and interpretation of the manuscript.

## Conflict of Interest

YP was employed by company the Shanghai Applied Protein Technology Co., Ltd. The remaining authors declare that the research was conducted in the absence of any commercial or financial relationships that could be construed as a potential conflict of interest.

## Footnotes

1 http://geneontology.org

## References

[B1] Aronsson K. Rönner U. Borch E. (2005). Inactivation of *Escherichia coli*, listeria innocua and saccharomyces cerevisiae in relation to membrane permeabilization and subsequent leakage of intracellular compounds due to pulsed electric field processing. *Int. J. Food Microbiol.* 99 19–32. 10.1016/j.ijfoodmicro.2004.07.012 15718026

[B2] Bolla J. R. (2014). *Bacterial Multidrug Efflux Pumps: Structure, Function and Regulation.* Ph.D. thesis, Iowa State University, Ames, IA.

[B3] Cebrián G. Condón S. Mañas P. (2016). Influence of growth and treatment temperature on staphylococcus aureus resistance to pulsed electric fields: relationship with membrane fluidity. *Innov. Food Sci. Emerg. Technol.* 37 161–169. 10.1016/j.ifset.2016.08.011

[B4] Chueca B. Pagán R. García-Gonzalo D. (2015). Transcriptomic analysis of *Escherichia coli* MG1655 cells exposed to pulsed electric fields. *Innov. Food Sci. Emerg. Technol.* 29 78–86. 10.1016/j.ifset.2014.09.003

[B5] Clark J. (2006). Pulsed electric field processing. *Food Technol.* 60 66–67.

[B6] Cox M. Hein C. Luber I. Paron N. Nagaraj N. Mann M. (2014). Accurate proteome-wide label-free quantification by delayed normalization and maximal peptide ratio extraction, termed maxlfq. *Mol. Cell. Proteomics* 13 2513–2526. 10.1074/mcp.M113.031591 24942700 PMC4159666

[B7] Cox M. Mann M. (2008). Maxquant enables high peptide identification rates, individualized p.p.b.-range mass accuracies and proteome-wide protein quantification. *Nat. Biotechnol.* 26 1367–1372. 10.1038/nbt.1511 19029910

[B8] Crowe J. H. Carpenter J. F. Crowe L. M. (1998). The role of vitrification in anhydrobiosis. *Annu. Rev. Physiol.* 60 73–103. 10.1146/annurev.physiol.60.1.73 9558455

[B9] Crowe L. M. (2002). Lessons from nature: the role of sugars in anhydrobiosis. *Comp. Biochem. Physiol. Part A Mol. Integr. Physiol.* 131 505–513. 10.1016/s1095-6433(01)00503-7 11867276

[B10] Dymek K. Dejmek P. Panarese V. António A. V. Wadsö L. Finnie C. (2012). Effect of pulsed electric field on the germination of barley seeds. *LWT Food Sci. Technol.* 47 161–166. 10.1016/j.lwt.2011.12.019

[B11] Esterbauer H. Schaur R. J. Zollner H. (1991). Chemistry and biochemistry of 4-hydroxynonenal, malonaldehyde and elated aldehydes. *Free Rad. Biol. Med.* 11 81–128. 10.1016/0891-5849(91)90192-6 1937131

[B12] García D. Mañas P. Gómez N. Raso J. Pagán R. (2010). Biosynthetic requirements for the repair of sublethal membrane damage in *Escherichia coli* cells after pulsed electric fields. *J. Appl. Microbiol.* 100 428–435. 10.1111/j.1365-2672.2005.02795.x 16478482

[B13] Gerhardt P. N. M. Tombras S. L. Smith G. M. (2000). Osmotic and chill activation of glycine betaine porter ii in listeria monocytogenes membrane vesicles. *J. Bacteriol.* 182 2544–2550. 10.1128/JB.182.9.2544-2550.2000 10762257 PMC111319

[B14] Goettel M. Eing C. Gusbeth C. Straessner R. Frey W. (2013). Pulsed electric field assisted extraction of intracellular valuables from microalgae. *Algal Res.* 2 401–408. 10.1016/j.algal.2013.07.004

[B15] Guerrero B. J. Á Welti C. J. (2016). “Pulsed electric fields,” in *Encyclopedia of Food and Health*, eds Caballero B. Finglas P. M. Toldrá F., (Cambridge, MA: Academic Press), 561–565. 10.1016/B978-0-12-384947-2.00579-1

[B16] Guillot A. Obis D. Mistou M. Y. (2000). Fatty acid membrane composition and activation of glycine-betaine transport in lactococcuslactis, subjected to osmotic stress. *Int. J. Food Microbiol.* 55 47–51. 10.1016/S0168-1605(00)00193-8 10791716

[B17] Guionet A. Joubert-Durigneux V. Packan D. Cheype C. Garnier J. P. David F. (2014). Effect of nanosecond pulsed electric field on *Escherichia coli* in water: inactivation and impact on protein changes. *J. Appl. Microbiol.* 117 721–728. 10.1111/jam.12558 24891291

[B18] Gustavsson N. Diez A. Nyström T. (2002). The universal stress protein paralogues of *Escherichia coli* are co-ordinately regulated and co-operate in the defence against dna damage. *Mol. Micobiol.* 43 107–117. 10.1046/j.1365-2958.2002.02720.x 11849540

[B19] Heller L. C. Cruz Y. L. Ferraro B. Yang H. Heller R. (2010). Plasmid injection and application of electric pulses alter endogenous mRNA and protein expression in B16. *F*10 mouse melanomas. *Cancer Gene Ther.* 17 864–871. 10.1038/cgt.2010.43 20706286 PMC2981654

[B20] Heng S. S. J. Chan O. Y. W. Keng B. M. H. Ling M. H. (2011). Glucan biosynthesis protein G is a suitable reference gene in *Escherichia coli* K-12. *ISRN Microbiol.* 2011:469053. 10.5402/2011/469053 23724305 PMC3658596

[B21] Hermawan N. Evrendilek G. A. Dantzer W. R. Zhang Q. H. Richter E. R. (2004). Pulsed electric field treatment of liquid whole egg inoculated with *Salmonella enteritidis*. *J. Food Saf.* 24 71–85. 10.1111/j.1745-4565.2004.tb00376.x

[B22] Kanehisa M. Sato Y. Furumichi M. Morishima K. Tanabe M. (2019). New approach for understanding genome variations in KEGG. *Nucleic Acids Res.* 47 D590–D595. 10.1093/nar/gky962 30321428 PMC6324070

[B23] La H. J. Choi G. G. Cho C. Seo S. H. Srivastava A. Jo B. H. (2015). Increased lipid productivity of Acutodesmus dimorphus using optimized pulsed electric field. *J. Appl. Phycol.* 28 931–938. 10.1007/s10811-015-0674-6

[B24] Lampe J. W. (1999). Health effects of vegetables and fruits: assessing mechanisms of action in human experimental studies. *Am. J. Clin. Nutr.* 70 475–490. 10.1016/j.molstruc.2008.02.018 10479220

[B25] Lazar C. Gatto L. Ferro M. Bruley C. Burger T. (2016). Accounting for the multiple natures of missing values in label-free quantitative proteomics data sets to compare imputation strategies. *J. Proteome Res.* 15 1116–1125. 10.1021/acs.jproteome.5b00981 26906401

[B26] Lebovka N. I. Bazhal M. I. Vorobiev E. I. (2001). Pulsed electric field breakage of cellular tissues: visualisation of percolative properties. *Innov. Food Sci. Emerg. Technol.* 2 113–125. 10.1016/S1466-8564(01)00024-8

[B27] Li J. Liao X. Zhong K. Zhang Y. (2011). Inactivation mechanism of pulsed electric fields on *Saccharomyces cerevisiae*. *Trans. Chin. Soc. Agric. Eng.* 27 355–360. 10.3969/j.issn.1002-6819.2011.04.062

[B28] Li W. (2016). Bringing bioactive compounds into membranes: the ubia superfamily of intramembrane aromatic prenyltransferases. *Trends Biochem. Sci.* 41 356–370. 10.1016/j.tibs.2016.01.007 26922674 PMC4911241

[B29] Lin S. Guo Y. You Q. Yin Y. Liu J. (2012). Preparation of antioxidant peptide from egg white protein and improvement of its activities assisted by high-intensity pulsed electric field. *J. Sci. Food Agric.* 92 1554–1561. 10.1002/jsfa.4742 22161302

[B30] Liu J. J. Kirti S. Luca Z. Chongguang C. Humphrey S. J. Yi-Ting C. (2018). In vivo brain gpcr signaling elucidated by phosphoproteomics. *Science* 360:eaao4927. 10.1126/science.aao4927 29930108 PMC6527112

[B31] Liu Z. Lv J. Zhang Z. Li H. Yang B. Chen W. (2019). Integrative transcriptome and proteome analysis identifies major metabolic pathways involved in pepper fruit development. *J. Proteome Res.* 18 982–994. 10.1021/acs.jproteome.8b00673 30650966

[B32] Liu Z. Y. Song Y. B. Guo Y. M. Wang H. T. Liu J. T. (2016). Optimization of pulsed electric field pretreatment parameters for preserving the quality of *Raphanus sativus*. *Drying Technol.* 34 692–702. 10.1080/07373937.2015.1070859

[B33] Liu Z. W. Han Z. Zeng X. A. Sun D. W. Aadil R. M. (2016). Effects of vesicle components on the electro-permeability of lipid bilayers of vesicles induced by pulsed electric fields (pef) treatment. *J. Food Eng.* 179 88–97. 10.1016/j.jfoodeng.2016.02.003

[B34] Liu Z. W. Zeng X. A. Ngadi M. Han Z. (2017). Effect of cell membrane fatty acid composition of *Escherichia coli* on the resistance to pulsed electric field (pef) treatment. *LWT Food Sci. Technol.* 76 18–25. 10.1016/j.lwt.2016.10.019

[B35] Locke B. R. Sato M. Sunka P. Hoffmann M. R. Chang J. S. (2006). Electrohydraulic discharge and nonthermal plasma for water treatment. *Ind. Eng. Chem. Res.* 45 882–905. 10.1021/ie050981u

[B36] Lu Y. Liu X. Shi S. Su H. Bai X. Cai G. (2012). Bioinformatics analysis of proteomic profiles during the process of anti-thy1 nephritis. *Mol. Cell. Proteomics* 11:M111.008755. 10.1074/mcp.M111.008755 22159597 PMC3322559

[B37] Luo H. Zhou D. Liu X. Nie Z. Quiroga-Sánchez D. L. Chang Y. (2016). Production of 3-Hydroxypropionic acid via the propionyl-CoA pathway using recombinant *Escherichia coli* strains. *PLoS One* 11:e0156286. 10.1371/journal.pone.0156286 27227837 PMC4882031

[B38] Ma J. Chen T. Wu S. Yang C. Bai M. Shu K. (2019). iProX: an integrated proteome resource. *Nucleic Acids Res.* 47 D1211–D1217. 10.1093/nar/gky869 30252093 PMC6323926

[B39] MacLean B. Tomazela D. M. Shulman N. Chambers M. Finney G. L. Frewen B. (2010). Skyline: an open source document editor for creating and analyzing targeted proteomics experiments. *Bioinformatics* 26 966–968. 10.1093/bioinformatics/btq054 20147306 PMC2844992

[B40] Mailloux R. J. Lemire J. Appanna V. D. (2010). Metabolic networks to combat oxidative stress in *Pseudomonas fluorescens*. *Antonie Van Leeuwenhoek* 99 433–442. 10.1007/s10482-010-9538-x 21153706

[B41] Mascher T. Helmann J. D. Unden G. (2006). Perception in bacterial signal-transducing histidine kinases. *Microbiol. Mol. Biol. Rev.* 70 910–938. 10.1128/MMBR.00020-06 17158704 PMC1698512

[B42] Menzel R. Roth J. (1981). Purification of the putA gene product. A bifunctional membrane-bound protein from *Salmonella typhimurium* responsible for the two-step oxidation of proline to glutamate. *J. Biol. Chem.* 256 9755–9761. 6270100

[B43] Mlakar V. Todorovic V. Cemazar M. Glavac D. Sersa G. (2009). Electric pulses used in electrochemotherapy and electrogene therapy do not significantly change the expression profile of genes involved in the development of cancer in malignant melanoma cells. *BMC Cancer* 9:299. 10.1186/1471-2407-9-299 19709437 PMC2745430

[B44] Moriya Y. Itoh M. Okuda S. Yoshizawa A. C. Kanehisa M. (2007). Kaas: an automatic genome annotation and pathway reconstruction server. *Nucleic Acids Res.* 35 W182–W185. 10.1093/nar/gkm321 17526522 PMC1933193

[B45] Nachin L. Nannmark U. Nystrom T. (2005). Differential roles of the universal stress proteins of *Escherichia coli* in oxidative stress resistance, adhesion, and motility. *J. Bacteriol.* 187 6265–6272. 10.1128/JB.187.18.6265-6272.2005 16159758 PMC1236625

[B46] Neidhardt F. C. Nyström T. (2010). Expression and role of the universal stress protein, uspa, of *Escherichia coli* during growth arrest. *Mol. Microbiol.* 11 537–544. 10.1111/j.1365-2958.1994.tb00334.x 8152377

[B47] Nogami T. Mizushima S. (1983). Outer membrane porins are important in maintenance of the surface structure of *Escherichia coli* cells. *J. Bacteriol.* 156 402–408. 10.1111/j.1365-2672.1983.tb02640.x 6311801 PMC215095

[B48] Ono K. Akaike T. Sawa T. Kumagai Y. Wink D. A. Tantillo D. J. (2014). Redox chemistry and chemical biology of h2s, hydropersulfides, and derived species: implications of their possible biological activity and utility. *Free Rad. Biol. Med.* 77 82–94. 10.1016/j.freeradbiomed.2014.09.007 25229186 PMC4258476

[B49] Pakhomova O. N. Khorokhorina V. A. Bowman A. M. Rodaitė-Riševičienė R. Saulis G. Xiao S. (2012). Oxidative effects of nanosecond pulsed electric field exposure in cells and cell-free media. *Arch. Biochem. Biophys.* 527 55–64. 10.1016/j.abb.2012.08.004 22910297 PMC3459148

[B50] Pereira C. S. Hünenberger P. H. (2008). Effect of trehalose on a phospholipid membrane under mechanical stress. *Biophys. J.* 95 3525–3534. 10.1529/biophysj.108.131656 18599628 PMC2553110

[B51] Pfaffl M. W. (2001). A new mathematical model for relative quantification in real-time RT–PCR. *Nucleic Acids Res.* 29:e45. 10.1093/nar/29.9.e45 11328886 PMC55695

[B52] Qian J. Y. Ma L. J. Wang L. J. Jiang W. (2016). Effect of pulsed electric field on structural properties of protein in solid state. *LWT Food Sci. Technol.* 74 331–337. 10.1016/j.lwt.2016.07.068

[B53] Qu H. Yu H. Gu R. Chen D. Chen X. Huang Y. (2018). Proteomics for studying the effects of L. *rhamnosus LV*108 against non-alcoholic fatty liver disease in rats. *RSC Adv.* 8 38517–38528. 10.1039/c8ra06771f PMC9090571 35559112

[B54] Reina L. D. Tony J. Z. Howard Z. Q. Yousef A. E. (1998). Inactivation of listeria monocytogenes in milk by pulsed electric field. *J. Food Prot.* 61 1203–1206. 10.4315/0362-028X-61.9.1203 9766078

[B55] Rivas A. Pina-Pérez M. C. Rodriguez V. S. Zuñiga M. Martinez A. Rodrigo D. (2013). Sublethally damaged cells of *Escherichia coli* by pulsed electric fields: the chance of transformation and proteomic assays. *Food Res. Int.* 54 1120–1127. 10.1016/j.foodres.2013.01.014

[B56] Serafini A. Tan L. Horswell S. Howell S. Greenwood D. J. Hunt D. M. (2019). Mycobacterium tuberculosis requires glyoxylate shunt and reverse methylcitrate cycle for lactate and pyruvate metabolism. *Mol. Microbiol* 112 1284–1307. 10.1111/mmi.14362 31389636 PMC6851703

[B57] Serpersu E. H. Kinosita K. Tsong T. Y. (1985). Reversible and irreversible modification of erythrocyte membrane permeability by electric field. *Biochim. Biophys. Acta* 812 779–785. 10.1016/0005-2736(85)90272-X 3970906

[B58] Shatalin K. Shatalina E. Mironov A. Nudler E. (2011). H2S: a universal defense against antibiotics in bacteria. *Science* 334 986–990. 10.1126/science.1209855 22096201

[B59] Somolinos M. García D. Mañas P. Condón S. Pagán R. (2008). Effect of environmental factors and cell physiological state on pulsed electric fields resistance and repair capacity of various strains of *Escherichia coli*. *Int. J. Food Microbiol.* 124 260–267. 10.1016/j.ijfoodmicro.2008.03.021 18455818

[B60] Sotelo G. K. A. Hamid N. Oey I. Noemi G. M. Ma Q. Leong S. Y. (2015). Effect of pulsed electric fields on the flavour profile of, red-fleshed sweet cherries (prunusavium var. stella). *Molecules* 20 5223–5238. 10.3390/molecules20035223 25806548 PMC6272343

[B61] Stefan G. Juan M. G. Terol J. Williams T. D. Nagaraj S. H. María J. N. (2008). High-throughput functional annotation and data mining with the blast2go suite. *Nucleic Acids Res.* 36 3420–3435. 10.1093/nar/gkn176 18445632 PMC2425479

[B62] Sun Q. Zhang N. Wang J. Cao Y. Li X. Zhang H. (2016). A label-free differential proteomics analysis reveals the effect of melatonin on promoting fruit ripening and anthocyanin accumulation upon postharvest in tomato. *J. Pineal Res.* 61 138–153. 10.1111/jpi.12315 26820691

[B63] Tan Z. Black W. Yoon J. M. Shanks J. V. Jarboe L. R. (2017). Improving *Escherichia coli* membrane integrity and fatty acid production by expression tuning of fadl and OmpF. *Microb. Cell Factor.* 16:38. 10.1186/s12934-017-0650-8 28245829 PMC5331629

[B64] Tanino T. Sato S. Oshige M. Ohshima T. (2012). Analysis of the stress response of yeast saccharomyces cerevisiae toward pulsed electric field. *J. Electrostat.* 70 212–216. 10.1016/j.elstat.2012.01.003

[B65] Thorgersen M. P. Downs D. M. (2009). Oxidative stress and disruption of labile iron generate specific auxotrophic requirements in *Salmonella enterica*. *Microbiology* 155 295–304. 10.1099/mic.0.020727-0 19118370 PMC6756756

[B66] Tkaczuk K. L. A Shumilin I. Chruszcz M. Evdokimova E. Savchenko A. Minor W. (2013). Structural and functional insight into the universal stress protein family. *Evol. Appl.* 6 434–449. 10.1111/eva.12057 23745136 PMC3673472

[B67] Toepfl S. Heinz V. Knorr D. (2007). High intensity pulsed electric fields applied for food preservation. *Chem. Eng. Process.* 46 537–546. 10.1016/j.cep.2006.07.011

[B68] Tretter L. Adam V. V. (2000). Inhibition of Krebs cycle enzymes by hydrogen peroxide: a key role of α-ketoglutarate dehydrogenase in limiting NADH production under oxidative stress. *J. Neurosci.* 20 8972–8979. 10.1523/JNEUROSCI.20-24-08972.2000 11124972 PMC6773008

[B69] Tyteca D. Schanck A. Y. Dufrêne F. Deleu M. Courtoy P. J. Tulkens P. M. (2003). The macrolide antibiotic azithromycin interacts with lipids and affects membrane organization and fluidity: studies on langmuir-blodgett monolayers, liposomes and j774 macrophages. *J. Membr. Biol.* 192 203–215. 10.1007/s00232-002-1076-7 12820665

[B70] Ulmer H. M. Heinz V. Gänzle M. G. Knorr D. Vogel R. F. (2002). Effects of pulsed electric fields on inactivation and metabolic activity of *Lactobacillus plantarum* in model beer. *J. Appl. Microbiol.* 93 326–335. 10.1046/j.1365-2672.2002.01699.x 12147082

[B71] Unal R. Yousef A. E. Dunne C. P. (2002). Spectrofluometric assessment of bacterial cell damage by pulsed electric field. *Innov. Food Sci. Emerg. Technol.* 3 247–254. 10.1016/S1466-8564(02)00033-4

[B72] Weaver J. C. Chizmadzhev Y. A. (1996). Theory of electroporation: a review. *Bioelectrochem. Bioenerg.* 41 135–160. 10.1016/s0302-4598(96)05062-3

[B73] Wu G. Gao H. (2017). Endogenous production and physiological functions of hydrogen sulfide in facultative anaerobic bacteria. *Acta Microbiol. Sin.* 57 170–178. 29750479

[B74] Wu V. C. H. (2008). A review of microbial injury and recovery methods in food. *Food Microbiol.* 25 735–744. 10.1016/j.fm.2008.04.011 18620965

[B75] Xue D. Farid M. M. (2015). Pulsed electric field extraction of valuable compounds from white button mushroom (*Agaricus bisporus*). *Innov. Food Sci. Emerg. Technol.* 29 178–186. 10.1016/j.ifset.2015.03.012

[B76] Yu Y. Jin T. Z. Xiao G. (2017). Effects of pulsed electric fields pretreatment and drying method on drying characteristics and nutritive quality of blueberries. *J. Food Process. Preserv.* 41:e13303 10.1111/jfpp.13303

[B77] Yun O. Zeng X. A. Brennan C. S. Han Z. Yun O. Zeng X. A. (2016). Effect of pulsed electric field on membrane lipids and oxidative injury of *Salmonella typhimurium*. *Int. J. Mol. Sci.* 17:1374. 10.3390/ijms17081374 27556460 PMC5000769

[B78] Zhang L. Alfano J. R. Becker D. F. (2015). Proline metabolism increases katG expression and oxidative stress resistance in *Escherichia coli*. *J. Bacteriol.* 197 431–440. 10.1128/JB.02282-14 25384482 PMC4285992

[B79] Zhao M. Zhao D. Ma Y. Hu Z. Wei Z. (2018). Quantitative proteomic analysis of cell responses to electroporation, a classical gene delivery approach. *Proteomics* 18:1800127. 10.1002/pmic.201800127 30035351

[B80] Zhou S. Yi T. Liu R. Bian C. Qi X. He X. (2012). Proteomics identification of annexin a2 as a key mediator in the metastasis and proangiogenesis of endometrial cells in human adenomyosis. *Mol. Cell. Proteomics* 11 3929–3936. 10.1074/mcp.M112.017988 22493182 PMC3394960

[B81] Zhu G. Cai G. Liu Y. Tan H. Yu C. Huang M. (2014). Quantitative itraqlc-ms/ms proteomics reveals transcription factor crosstalk and regulatory networks in hypopharyngeal squamous cell carcinoma. *J. Cancer* 5 525–536. 10.7150/jca.9207 24963357 PMC4067512

[B82] Zimmermann U. Pilwat G. Riemann F. (1974). Dielectric breakdown of cell membranes. *Biophys. Struct. Mech.* 14 881–899. 10.1016/S0006-3495(74)85956-4 PMC1334582 4611517

